# Preadmission Testing in the Context of Social Determinants of Health: A Narrative Review of Gaps, Challenges, and Opportunities for Equitable Perioperative Care

**DOI:** 10.7759/cureus.94200

**Published:** 2025-10-09

**Authors:** Lawrence W Chinn, Luis Cupertino

**Affiliations:** 1 Anesthesiology and Perioperative Medicine, Rutgers University New Jersey Medical School, Newark, USA

**Keywords:** digital divides, health equity in perioperative care, housing instability, limited english proficiency, low health literacy, perioperative disparities, preadmission testing (pat), social determinants of health (sdoh), surgical risk stratification, transportation barriers

## Abstract

Preadmission testing (PAT) represents a critical step in perioperative care, designed to optimize readiness and minimize same-day cancellations, yet its traditional model assumes patients can reliably complete laboratory testing, specialist consultations, and preoperative instructions without structured support. A growing body of evidence demonstrates that social determinants of health (SDOH), including limited health literacy, language barriers, transportation difficulties, housing instability, digital access inequities, and work or caregiving responsibilities, directly affect patient readiness and outcomes. Studies confirm that transportation costs, housing insecurity, and unstable caregiving arrangements increase missed appointments, while patients with limited English proficiency or low health literacy are at greater risk of miscommunication, misunderstanding instructions, and adverse perioperative outcomes. Digital divides further undermine the potential benefits of virtual PAT models, with lower-income and older patients less likely to access or use video-based visits. Interventions such as frailty screening, patient navigation, professional interpreter integration, plain-language education, e-health modules, and structured prehabilitation programs have shown promise in improving comprehension, satisfaction, readmissions, and even survival. However, these approaches remain inconsistently implemented and often limited to single-center initiatives. This review synthesizes current literature linking SDOH to perioperative readiness and highlights evidence-based strategies that can be integrated into PAT workflows. Embedding equity-focused screening, advocacy, flexible scheduling, hybrid digital options, and tailored education into PAT can help transform it from a potential source of inequity into a consistent gateway for safer, more inclusive surgical care.

## Introduction and background

Preadmission testing (PAT), the structured preoperative process that ensures patients are optimized and ready for surgery, remains a longstanding pillar of modern perioperative practice. It is designed to identify modifiable comorbidities, order appropriate laboratory tests, coordinate consultations, and educate patients to ensure safe surgical care. The goal is to reduce day-of-surgery complications, prevent delays, and promote efficiency in operating room scheduling. Guidelines by the American Society of Anesthesiologists (ASA) and other professional societies have progressively shifted from routine universal testing to risk-stratified approaches based on patient history and type of surgery [1,2].

Yet even as clinical algorithms become more nuanced, perioperative pathways often assume patients have stable housing, transportation, reliable communication channels, sufficient literacy, English proficiency, and access to digital technologies. For many patients, these assumptions do not hold. National surveys indicate that nearly one-third of US adults have limited health literacy, which undermines their ability to follow complex perioperative instructions [3]. Limited English proficiency (LEP) has been independently associated with worse surgical outcomes and higher rates of perioperative complications, largely due to communication errors and inconsistent interpreter access [4-6]. These findings align with recent systematic reviews demonstrating that LEP is an independent risk factor for delays, longer lengths of stay, and discharge to facilities rather than home. These literacy and language factors are part of the broader social determinants of health (SDOH) that shape readiness for surgery.

Transportation and housing instability present additional barriers. Patients lacking reliable transportation are more likely to miss preoperative appointments, while those without stable housing face challenges executing instructions such as bowel preparations or medication adjustments [7]. As virtual and telehealth-based PAT pathways expand, digital access gaps among older, low-income, and rural patients emerge as another barrier, excluding those without broadband or digital literacy [8,9].

These inequities translate directly into perioperative outcomes. Patients facing adverse SDOH are more likely to experience incomplete preoperative clearance, last-minute cancellations, and potentially avoidable perioperative risk. Reviews and observational studies confirm that social risk factors, including low socioeconomic status (SES) and LEP, contribute to higher surgical complication rates and worse patient-centered outcomes [10-12]. Health systems bear both the financial and logistical burdens of these disruptions, while patients face delays and marginalization when they cannot complete PAT steps as designed. Despite this, the perioperative literature has only recently begun to address equity gaps in preoperative evaluation systematically. This review, therefore, examines how key SDOH domains intersect with the PAT process and identifies evidence-based strategies to promote equity in perioperative care.

Methods

This narrative review was conducted to synthesize current knowledge on how SDOH influence PAT and perioperative outcomes. A targeted literature search of PubMed was performed for English-language articles published between January 2000 and June 2025, following a structured narrative approach designed to enhance transparency and reproducibility. Search terms combined concepts related to PAT and perioperative care with specific SDOH domains.

For the perioperative scope, terms included “preadmission testing,” “pre-admission testing,” “preoperative assessment,” “preoperative evaluation,” “preoperative clinic,” “preanesthesia evaluation,” “perioperative care,” “anesthesiology,” and “surgery.” To capture SDOH domains, terms included “social determinants of health,” “health equity,” “healthcare disparities,” “socioeconomic factors,” “transportation barriers,” “housing instability,” “homelessness,” “health literacy,” “limited English proficiency,” “language barriers,” “interpreter,” “digital divide,” “telehealth,” “telemedicine,” “patient portal,” “caregiver,” “frailty,” “prehabilitation,” “patient navigation,” “advocacy,” and “indigenous” or “low- and middle-income countries.” Additional search concepts targeted preoperative education and outcomes, including “teach-back,” “plain language,” “patient education,” “video education,” “web-based,” “cancellation,” “operating room delay,” “readmission,” “length of stay,” “discharge destination,” “post-acute care,” “transfusion,” “mortality,” and “patient-reported outcome measures.”

Articles were screened for relevance to PAT or perioperative workflows. Studies were included if they examined how one or more SDOH domains impacted surgical readiness, perioperative processes, or patient outcomes. Priority was given to guidelines, systematic reviews, cohort studies, and interventional reports describing strategies to mitigate inequities. Reference lists of included studies were also reviewed to identify additional relevant publications.

Both authors independently screened titles and abstracts for relevance, followed by a full-text review of potentially eligible studies. Disagreements were resolved by consensus. Studies were included if they evaluated how one or more SDOH domains influenced PAT, preoperative readiness, perioperative processes, or surgical outcomes. Priority was given to guidelines, systematic reviews, cohort, and interventional studies with clear methodological rigor. Non-perioperative studies, case reports, and commentaries without data were excluded. These steps were undertaken to minimize selection bias and strengthen the validity of the narrative synthesis.

Because of heterogeneity in study designs, populations, and outcomes, no quantitative meta-analysis or pooled statistical testing was performed. Instead, findings were synthesized descriptively, emphasizing convergence across study types and consistency of themes rather than formal effect size estimation.

## Review

The search and supplementary identification steps yielded a heterogeneous body of 73 included sources spanning professional guidelines, systematic and scoping reviews, observational cohorts, interventional trials, and policy or position pieces relevant to SDOH in perioperative care. The synthesis incorporated several recent studies published between 2023 and 2025 to ensure that the findings reflect current evidence in perioperative equity. Across the structured narrative synthesis, several recurring domains with direct implications for PAT were identified.

The subsections that follow focus on patient- and process-level domains most directly influenced by preadmission workflows, while macro-level determinants such as race, insurance coverage, and systemic inequities are analyzed later in the manuscript.

Transportation and housing instability remain among the most tangible barriers to surgical readiness. Patients who lack reliable transportation or live far from the hospital often struggle to complete required laboratory testing, imaging, or consultations before surgery. Those facing unstable housing may find it difficult to store medications, follow preparation instructions, or maintain consistent communication with the care team. When travel costs or logistical challenges are reduced, attendance rates generally improve, illustrating how sensitive in-person PAT requirements are to practical access barriers.

Health literacy also strongly influences a patient’s ability to prepare for surgery. Individuals with difficulty understanding medical instructions may misinterpret fasting or medication directions, leading to delays or cancellations. Clear communication, repetition, and plain-language education can substantially improve comprehension and confidence, ensuring that patients are ready for surgery and understand what to expect before and after the procedure.

Language and communication challenges further compound these issues. Patients who do not share the same primary language as their medical team are more likely to misunderstand instructions or receive incomplete preparation. Access to professional interpretation and bilingual educational materials can help prevent errors, enhance satisfaction, and support safe preparation for surgery. When these services are inconsistent or unavailable, even well-designed preadmission programs can fail to reach those who most need them.

Digital access has become another important determinant of readiness as telehealth and virtual preoperative visits become more common. Not all patients have broadband internet, suitable devices, or the digital literacy needed to participate effectively in virtual assessments. Hybrid models that combine digital options with telephone or in-person visits can help close this gap and ensure that patients remain engaged regardless of technology access or comfort level.

Work and family responsibilities also influence a patient’s ability to complete PAT. Many individuals must balance caregiving duties, employment obligations, and transportation challenges, leaving little flexibility for daytime clinic appointments. Without accommodations such as extended hours or coordinated scheduling, these competing demands can lead to missed appointments and incomplete preparation. Older adults without reliable social support face additional obstacles in organizing transportation, understanding instructions, or managing preoperative steps on their own.

Taken together, these interconnected social and logistical factors demonstrate that readiness for surgery is shaped by far more than medical optimization alone. Addressing these realities within PAT programs is essential to achieving equitable and efficient perioperative care.

The traditional model and its assumptions

Building on the identified SDOH domains, it becomes clear that many of the barriers affecting surgical readiness arise from assumptions embedded in the traditional PAT model itself. In most institutions, PAT is structured around a standardized sequence: a medical history and risk assessment interview, laboratory and imaging studies when indicated, specialist consultations as required, and patient education about perioperative instructions. This model was developed to optimize readiness and prevent day-of-surgery delays [1]. However, it rests on the assumption that once patients are scheduled for surgery, they can reliably complete all recommended steps without additional support.

In reality, the assumption that patients will seamlessly complete every step of PAT often fails under the weight of social barriers. A 2023 review summarizes growing evidence that social vulnerability, including economic disadvantage and unstable living conditions, shapes surgical access, recovery, and resource use [11]. These findings are consistent with earlier chronic disease literature but extend it into surgical populations, highlighting that similar inequities directly compromise perioperative readiness. For patients with LEP, recent multicenter work shows that interpreter services are inconsistently provided (only 53% in the first 24 hours), 3.4% had interpreter use documented at discharge, and just 12% received language-concordant discharge forms, conditions that jeopardize safe preparation and follow-up [12]. This expands on prior single-center reports by providing system-level confirmation that language barriers remain inadequately addressed. Older adults with frailty describe additional barriers, including transportation challenges, caregiver constraints, and difficulty attending multiple appointments, which reduce participation in optimization and prehabilitation efforts essential for safe surgery [13]. Together, these findings emphasize that preadmission workflows designed on assumptions of patient self-sufficiency risk systematically excluding those already at the highest risk for poor perioperative outcomes.

In recent years, some centers have introduced virtual and telephone-based PAT to improve efficiency, particularly during the COVID-19 pandemic. While promising, these models can widen inequities when patients lack reliable broadband, digital literacy, or a stable phone connection [9]. For example, a Frontiers survey found that nearly one-quarter of caregivers of pediatric patients reported being affected by the digital divide, difficulty with devices, internet, or comfort using health apps, which can limit their ability to follow preoperative instructions delivered virtually [14]. In addition, during the COVID-19 response, video-telehealth services were more likely to be used by younger, higher-income, and more educated patients, whereas older, lower SES, or less digitally literate individuals more often relied on audio-only calls, reducing the fidelity and interactivity of communication [15]. Telehealth prehabilitation programs for cancer patients in rural areas were well-received, but qualitative feedback identified logistical barriers, such as connectivity and comprehension, that limited full engagement among some participants [16]. Together, these studies indicate that while telehealth can expand reach, its benefits are unevenly distributed and may reinforce disparities without deliberate design.

Structured education further illustrates how assumptions of patient self-sufficiency can fail. Studies of preadmission education demonstrate that structured preparation improves patient understanding and lowers anxiety, underscoring that “self-directed” navigation of instructions is insufficient for many patients [17]. Similarly, socioeconomic disadvantage predicts delays and deviations from guideline-adherent perioperative care, including in head and neck cancer patients, where race, insurance, and income disparities drive postoperative radiotherapy delays [18]. These findings expand upon prior general equity research by showing how SDOH specifically affect preoperative pathways. When patients are unable to meet the expectations of the traditional PAT model, inefficiencies accumulate. Evidence shows that incomplete preoperative assessment contributes to operating room cancellations and delays, wasting both institutional resources and patient opportunities for timely care [19]. This illustrates how social risk factors can translate directly into system inefficiency within perioperative workflows. Together, these studies highlight how PAT systems designed for socially advantaged populations can inadvertently reinforce disparities if they do not systematically account for diverse patient needs.

How specific SDOH factors intersect with PAT

Transportation and Housing Instability

Transportation and housing insecurity remain central barriers to successful preoperative preparation. For example, in a low-resource international setting, removing transportation costs reduced surgical no-show rates by about half, showing the payment burden alone can prevent patients from completing surgical appointments [20]. These findings are consistent with earlier US data showing that transportation barriers broadly limit access to outpatient and preoperative care [7]. Within the US, about one in three frequent healthcare users (publicly insured/low-income) report transportation barriers that lead to missed or delayed medical appointments, including for surgical or preoperative care [21]. Housing instability likewise undermines readiness. Patients who are unhoused or living in unstable housing conditions are more likely to miss surgical appointments, have higher rates of comorbidities, experience increased postoperative complications, face longer hospitalizations, and encounter less predictable discharge patterns [22]. Qualitative work from surgical providers confirms that homelessness impacts multiple phases of care, from preoperative evaluation through postoperative follow-up, due to a lack of stable contact info, transportation, and living environment for recovery [23]. Travel time to hospitals also matters. In surgical emergencies, patients residing more than 60 minutes from treatment facilities present with more advanced disease, are more likely to require interfacility transfers, and often experience prolonged admissions [24]. For PAT workflows, these findings imply that preadmission steps depending on physical presence (labs, imaging, specialist consults) are especially vulnerable to disruption among those with unstable housing, no vehicle, or long travel times [20-24].

Health Literacy and Comprehension

Low health literacy impairs the ability to follow complex perioperative instructions, from fasting requirements to anticoagulant management. Recent evidence shows that among elective surgery patients in the Netherlands, about 37% have limited health literacy, with particularly low scores in judgment and appraisal of information, domains critical for understanding complex instructions like medication changes or procedural logistics [25]. A systematic review encompassing over 1,100 surgical patients found that poor health literacy is strongly associated with worse understanding of both operative procedures and discharge instructions, and reduced compliance with preoperative instructions such as fasting or medication discontinuation [26]. These findings align with broader health services research but specifically extend the evidence into perioperative pathways, where misinterpretation can translate into cancellation or increased complication risk [3]. In a more focused outcome, patients with lower health literacy have also been shown to recall postoperative instructions less accurately, particularly in orthopedic surgical populations, raising the risk of complications or readmissions [27]. Structured web-based education tools provide promising mitigation: a recent day surgery web intervention improved understanding of surgical preparation among patients in a hand surgery cohort [28]. These results suggest that digital education, when designed at accessible reading levels, has the potential to close comprehension gaps in targeted populations. More broadly, reviews emphasize that addressing literacy within the preoperative setting is a core component of equity-focused care, urging perioperative teams to integrate structured education, plain-language counseling, and standardized comprehension checks into PAT workflows [29,30]. These findings underscore that literacy challenges are not abstract risks but active drivers of perioperative inequity, and that PAT workflows must include plain language, repetition, comprehension checks, and accessible formats to reach at-risk populations effectively.

Language and Communication

Language barriers compound these issues by undermining accurate communication and placing LEP patients at risk of misunderstanding or incomplete preparation. Recent cohort data show that while many surgical patients with LEP receive interpreter support at some point during hospitalization, only about half receive interpreter services early, fewer than 5% receive interpreter services at discharge, and 12% are given language-concordant discharge paperwork, leaving critical instructions vulnerable to misinterpretation [12]. Systematic reviews reinforce that LEP is associated with poorer understanding of procedural consent, worse pain control postoperatively, and misunderstandings of discharge instructions when professional interpreters are not consistently used [6]. Another review found that LEP patients face delays in access, longer hospital stays, and are more frequently discharged to skilled care facilities, outcomes tied to communication gaps across the perioperative process [10]. System-level surveys reveal that variability in availability of professional medical interpreter (PMI) services, inconsistent documentation, and reliance on ad-hoc interpreters (staff, family) persist in many centers, increasing risk for LEP patients of adverse surgical outcomes [31]. Evidence also shows improvements in patient satisfaction and comprehension when consistent interpreter services are employed, suggesting embedding PMI and communication advocacy into PAT workflows is essential-not optional [32]. Emerging work on patient advocacy in perioperative settings highlights that patients with LEP often rely on ad hoc communication strategies, leaving them vulnerable to misunderstanding or incomplete preparation [33]. PAT processes that assume language concordance risk reinforcing disparities unless professional interpretation and advocacy are embedded in the model.

Digital Access

As virtual preoperative assessment becomes more common, access to digital infrastructure has become a new determinant of perioperative readiness. A global review of perioperative pathways in low- and middle-income countries (LMIC) underscores how technology access and health system resources shape the feasibility of virtual preoperative care [34]. However, in many settings, patients simply do not have stable broadband, devices, or digital literacy to make virtual PAT meaningful. For example, Choxi et al. (2022) identified that many ambulatory care patients lack high-speed internet or devices suitable for video visits, significantly limiting access [35]. Cheng et al. (2023) found that lower eHealth literacy correlates with reduced engagement in video telehealth among hospitalized populations, even when telehealth is offered [36]. In rural surgical populations, studies of tele-prehabilitation show strong interest, but uptake and retention are hindered by connectivity problems or lack of device access [16]. In one redesign of pre-anesthesia evaluation, digital patient questionnaires and patient portal education were implemented with success, but patient satisfaction and cancellation rates still depended in part on how well patients could use or access the digital tools [37]. Among caregivers of pediatric patients, nearly a quarter report at least one barrier to access, familiarity, or readiness to use digital health applications, suggesting that the “digital by default” model risks leaving behind those without digital comfort or devices [14]. In low-income settings, similar divides persist for patients without reliable broadband or device access, raising concerns that defaulting to tele-PAT could systematically exclude vulnerable populations.

Work and Family Responsibilities

Caregiving demands and employment obligations can also interfere with patients’ ability to complete multiple appointments or adhere to rigid schedules. Evidence from pediatric orthopedic clinics shows that patients with greater socioeconomic risk, including caregiver employment constraints, have significantly higher rates of missed preoperative or clinic appointments [38]. Unpaid caregivers frequently report needing to juggle medical obligations for family members with work shifts, transportation, and other responsibilities, sometimes foregoing necessary medical visits or optimization steps due to lack of flexibility or support [39]. Delays in surgical care, from scheduling through preoperative evaluation, have been shown to disproportionately burden patients with less workplace flexibility or those without backup caregivers, amplifying anxiety, financial hardship, and risk of incomplete readiness [40]. Evidence from perioperative equity studies in older adults demonstrates that those with limited social support experience greater difficulty navigating preoperative care [41]. In pediatrics, disparities in perioperative pain management and broader SDOH challenges emphasize how caregiver capacity directly shapes a child’s readiness for surgery [42,43]. Moreover, social determinants interact with medical complexity to influence not only perioperative readiness but also long-term outcomes, as demonstrated in children with congenital heart diseases where neurodevelopmental impairments persist despite advances in surgical care [44]. These findings suggest that work and family responsibilities must be explicitly recognized in PAT design.

Consequences of unaddressed SDOH in PAT

When social risk is not addressed within preadmission workflows, patients are more likely to arrive incompletely prepared, experience preventable perioperative complications, and follow less favorable recovery trajectories [45-47]. Disparities tied to neighborhood deprivation, insurance/SES, and hospital context manifest as different discharge destinations and long-term outcomes after elective surgery, indicating that readiness gaps are not just clinical but structural [48]. Pediatric and maternal populations show similar patterns, with inequities in perioperative care and severe morbidity persisting despite protocolized pathways, underscoring that standardized care alone does not neutralize social risk [49]. Similar patterns are observed in oncology, where inadequate preoperative evaluation and missed follow-up, often exacerbated by social risk, directly contribute to preventable complications and delayed treatment [50].

At the systems level, social risk contributes to variation in resource use and practice patterns, such as post-acute spending after cardiac surgery and transfusion variability in coronary artery bypass grafts, signaling inefficiencies that can compound inequities for disadvantaged patients [51,52]. Facility context matters too: differences between safety-net and private hospitals in preoperative characteristics and outcomes highlight how organizational factors can amplify or attenuate patient-level social risk [53]. Collectively, overlooking SDOH in PAT increases cancellations and care delays, erodes quality and safety, and consumes scarce perioperative resources, costs borne most heavily by patients already facing structural barriers [54-56].

Opportunities for improvement

Build Communication and Advocacy Into PAT

Formalized advocacy pathways in acute/perioperative settings can mitigate literacy, cultural, and communication mismatches that otherwise lead to incomplete preparation; embedding advocacy roles and escalation routes within PAT helps close these gaps [57]. Specialty guidance offers pragmatic adaptations for preoperative evaluation in populations facing incarceration history, homelessness, and other social barriers, tools that can be operationalized as PAT checklists and referral scripts [58]. Advocacy is especially critical for older adults, where perioperative considerations such as comorbidity burden, frailty, and limited social support complicate preparation and recovery. Reviews in geriatric surgery highlight the importance of proactive planning and tailored perioperative assessment to ensure that vulnerable older patients are not disadvantaged by standard workflows [59]. Similarly, sex-based differences in prescribing patterns following arthroplasty illustrate how communication failures and implicit bias can translate into disparities in recovery, reinforcing the need for standardized advocacy and equitable dialogue during the PAT process [60].

Importantly, several health systems have already demonstrated that embedding structured screening and advocacy into preoperative pathways can improve outcomes. A multicenter frailty-screening initiative in the US linked electronic alerts to optimization pathways and showed significant reductions in one-year mortality among frail surgical patients once presurgical interventions were triggered [61]. Similarly, implementation of structured frailty screening with targeted optimization (nutrition, anesthesia review, social work support) reduced 30-day readmissions and trends toward fewer non-home discharges after vascular surgery [62]. These examples suggest that PAT programs adopting social risk and frailty screening with referral processes could achieve parallel improvements.

Looking ahead, these principles could be translated into practical process improvements. PAT programs might standardize a social risk screen at intake that flags patients with housing insecurity, LEP, or caregiver strain, prompting automatic referral to navigators or social work before surgery day. Checklists could incorporate structured prompts for interpreter engagement or require documentation that discharge instructions were provided in the patient’s preferred language. For older adults and those with functional limitations, PAT visits could embed frailty screening linked to tailored pathways, such as referral to geriatric assessment or targeted prehabilitation. For patients balancing employment or caregiving, PAT scheduling could expand to after-hours or weekend slots, reducing missed visits caused by rigid clinic hours. Finally, structured education could be delivered in layered formats (written, digital, and in-person teach-back), ensuring patients have multiple avenues to clarify key instructions. None of these changes requires a sweeping system overhaul, but together they represent a deliberate shift toward designing PAT pathways that recognize and accommodate diverse social contexts.

Standardize Equitable Education Delivery

Structured preadmission education and literacy-sensitive counseling improve knowledge, reduce anxiety, and increase adherence; randomized and pilot data support video/e-health modules as scalable adjuncts when paired with plain-language scripts and hybrid in-person options [63-65]. Beyond small pilots, larger randomized studies now demonstrate that e-health platforms can meaningfully improve engagement and patient-perceived equity of care. The One-4-ALL initiative confirmed that structured perioperative education via e-health improved comprehension and satisfaction across diverse patient populations, validating the role of digital tools as a core complement to traditional PAT education [66].

Design for Digital Constraints

Lessons from programs serving indigenous and remote communities show that telehealth improves reach only when infrastructure, language services, and in-person fallback options are planned upfront, arguing for hybrid PAT designs rather than “virtual-by-default” models [67]. Embedding options for interpreter-facilitated calls, device support, and fallback in-person visits ensures that digital-first pathways do not widen the very gaps they are intended to close.

Extend Readiness With Targeted Prehabilitation and Social-Support Alignment

**Figure 1:**
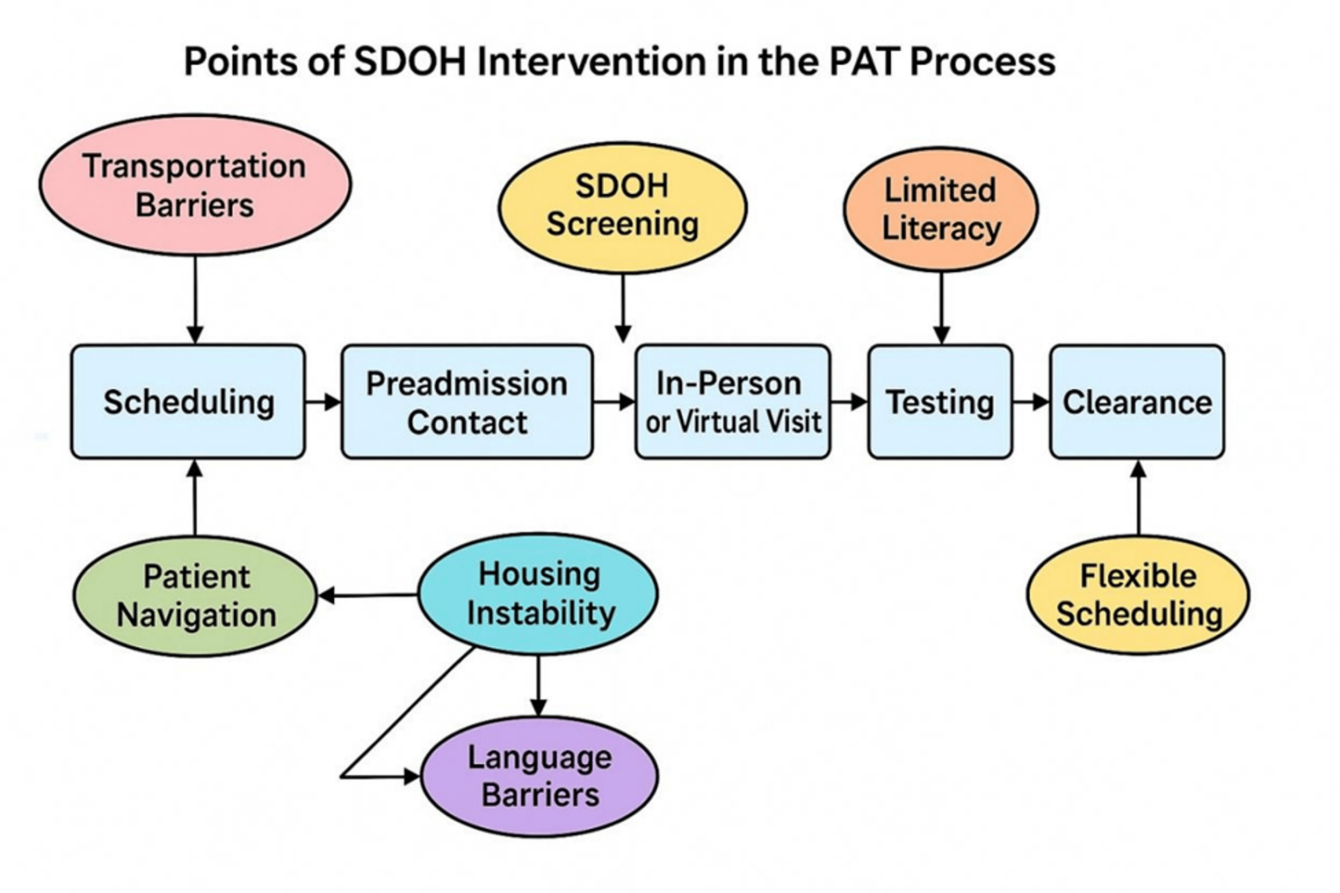
The figure illustrates how specific SDOH barriers intersect with key steps in the PAT process and highlights potential points for targeted intervention. Integration of social determinants of health (SDOH) into the preadmission testing (PAT) process. The figure illustrates where common barriers such as transportation, housing instability, health literacy, language, digital access, and competing responsibilities can interfere with readiness, and highlights potential points for targeted intervention. Image Credit: Lawrence W. Chinn, MD.

Patients with lower literacy and socioeconomic disadvantage are least likely to engage in prehabilitation despite higher potential benefit; aligning scheduling, transport, and caregiver availability can improve equitable uptake [68]. Evidence from orthopedic populations shows that structured preoperative interventions can reduce downstream healthcare use and improve return-to-work, underscoring the broader value of prehabilitation in perioperative equity efforts [69]. Similar findings emerge from fracture care, where immobilization strategies significantly influenced recovery quality [70], and from trials of early versus delayed weight bearing, which showed that rehabilitation timing directly altered long-term function [71]. Together, these data reinforce that without systematic prehabilitation referrals and standardized guidance, patients remain exposed to wide variation in recovery trajectories, an equity gap that PAT programs are uniquely positioned to address. Figure 1 illustrates how these and other interventions could be systematically integrated into each step of the PAT process to address SDOH barriers.

In addition to the workflow model shown in Figure 1, Table 1 summarizes common barriers and practical strategies that perioperative programs can adapt to address them.

**Table 1:** Common social determinants of health (SDOH) barriers affecting preadmission testing (PAT) and potential solutions. Social determinants of health (SDOH) barriers affecting preadmission testing (PAT), their impact on the completion of preoperative requirements, and evidence-informed solutions that perioperative programs can adapt to improve equity and readiness.

SDOH barrier	Impact on PAT	Proposed solutions
Transportation barriers	Missed labs, consults, workups	Rideshare; navigation; mobile phlebotomy
Housing instability	Prep/storage issues; med mismanagement	Shelter links; same-day testing; community outreach
Low health literacy	Misunderstood instructions, prep errors	Plain-language materials, teachback, tailored education
Limited English proficiency	Communication errors	Interpreters, multilingual handouts, bilingual staff
Digital divide	Missed telehealth visits	Hybrid visit, phone follow-up, digital literacy assistance
Responsibilities	No-shows, care conflicts, income loss	Flex hours, navigation, evening/weekend options

Research and policy implications

Closing equity gaps in PAT will require deliberate study designs that move beyond descriptive disparities to implementation frameworks. Work on thoracic surgery emphasizes that SDOH must be systematically assessed and integrated into practice guidelines, making perioperative research an essential vehicle for change [72]. Specialty reviews in anesthesiology underscore the persistence of racial and ethnic disparities and call for standardized data capture to stratify outcomes by social risk, which can inform quality reporting and reimbursement models [73].

Policy work should also align perioperative programs with international efforts. Reports from LMIC contexts and global initiatives in children’s surgery highlight that equitable perioperative access is now seen as a core health system priority, not an optional adjunct [56]. Embedding reimbursement for navigation, interpreter services, and prehabilitation, while tracking equity-focused outcomes such as cancellation rates, readmissions, and patient-reported outcome measures, will be necessary to sustain adoption. Research must focus on prospective designs that measure both clinical outcomes and patient-reported experiences, particularly in disadvantaged groups.

Limitations

This review has important limitations. It was designed as a structured narrative review rather than a systematic review, and although the PubMed search strategy captured a wide range of relevant studies, grey literature and non-indexed sources may have been missed. Both authors independently screened and reviewed studies to enhance validity, yet some risk of selection bias remains. Several included investigations relied on observational data, introducing potential confounding, while others reflected international or pediatric contexts that may not generalize directly to all PAT settings. Institutional practice variation also limits uniform applicability across programs. Nonetheless, the convergence of findings across multiple populations, designs, and health systems strengthens confidence that SDOH exert a consistent and clinically meaningful impact on preoperative readiness.

## Conclusions

As perioperative medicine continues to evolve, equity must remain a central principle. PAT is uniquely positioned to identify SDOH that influence surgical readiness, yet traditional models have often assumed that patients can easily follow instructions, access transportation, and manage competing responsibilities without structured support. This review demonstrates that these assumptions are frequently unrealistic and that overlooking such barriers can directly affect perioperative safety.

Moving forward, PAT can serve as a gateway to more equitable surgical care by embedding social-risk screening, interpreter services, patient navigation, and flexible education strategies into standard workflows. These approaches represent practical, evidence-supported adjustments that do not require major system redesign. By aligning PAT processes with equity goals, perioperative programs can reduce cancellations, improve patient comprehension, and ensure safer, more inclusive care for all surgical patients.

## References

[1] Apfelbaum JL, Connis RT, Nickinovich DG (2012). Practice advisory for preanesthesia evaluation: an updated report by the American Society of Anesthesiologists Task Force on Preanesthesia Evaluation. Anesthesiology.

[2] Thompson A, Fleischmann KE, Smilowitz NR (2024). 2024 AHA/ACC/ACS/ASNC/HRS/SCA/SCCT/SCMR/SVM guideline for perioperative cardiovascular management for noncardiac surgery: a report of the American College of Cardiology/American Heart Association Joint Committee on Clinical Practice Guidelines. J Am Coll Cardiol.

[3] Berkman ND, Sheridan SL, Donahue KE, Halpern DJ, Crotty K (2011). Low health literacy and health outcomes: an updated systematic review. Ann Intern Med.

[4] Flores G (2006). Language barriers to health care in the United States. N Engl J Med.

[5] Divi C, Koss RG, Schmaltz SP, Loeb JM (2007). Language proficiency and adverse events in US hospitals: a pilot study. Int J Qual Health Care.

[6] Luan-Erfe BM, Erfe JM, DeCaria B, Okocha O (2023). Limited English proficiency and perioperative patient-centered outcomes: a systematic review. Anesth Analg.

[7] Syed ST, Gerber BS, Sharp LK (2013). Traveling towards disease: transportation barriers to health care access. J Community Health.

[8] (2025). Pew Research Center. Internet, broadband fact sheet. https://www.pewresearch.org/internet/factsheet/internet-broadband/.

[9] Romero CS, Filipovic MG, Luedi MM (2024). Beyond the consulting room and telemedicine: unveiling the future of anesthesiology with virtual preoperative assessment. Anesthesiol Clin.

[10] Joo H, Fernández A, Wick EC, Moreno Lepe G, Manuel SP (2023). Association of language barriers with perioperative and surgical outcomes: a systematic review. JAMA Netw Open.

[11] Devin CL, Shaffer VO (2023). Social determinants of health and impact in perioperative space. Clin Colon Rectal Surg.

[12] Cevallos J, Lee C, Bongiovanni T (2024). Use of professional interpreters for patients with limited English proficiency undergoing surgery. JAMA Netw Open.

[13] Barnes K, Hladkowicz E, Dorrance K (2023). Barriers and facilitators to participation in exercise prehabilitation before cancer surgery for older adults with frailty: a qualitative study. BMC Geriatr.

[14] Claudio MC, Rehany Z, Stachtari K, Guadagno E, Osmanlliu E, Poenaru D (2024). Exploring the digital divide: results of a survey informing mobile application development. Front Digit Health.

[15] Buis LR, Brown LK, Plegue MA (2023). Identifying inequities in video and audio telehealth services for primary care encounters during COVID-19: repeated cross-sectional, observational study. J Med Internet Res.

[16] Waterland JL, Chahal R, Ismail H, Sinton C, Riedel B, Francis JJ, Denehy L (2021). Implementing a telehealth prehabilitation education session for patients preparing for major cancer surgery. BMC Health Serv Res.

[17] Zhang LH, Ying YF, Yin J, Li N, Cheng Y, Yu RY (2024). Effect of pre-admission "quasi-collective" education on health education for patients with ophthalmic day surgery. Technol Health Care.

[18] Duckett KA, Kassir MF, Nguyen SA (2024). Factors associated with head and neck cancer postoperative radiotherapy delays: a systematic review and meta-analysis. Otolaryngol Head Neck Surg.

[19] Ferschl MB, Tung A, Sweitzer B, Huo D, Glick DB (2005). Preoperative clinic visits reduce operating room cancellations and delays. Anesthesiology.

[20] Shrime MG, Hamer M, Mukhopadhyay S (2017). Effect of removing the barrier of transportation costs on surgical utilisation in Guinea, Madagascar and the Republic of Congo. BMJ Glob Health.

[21] Cochran AL, McDonald NC, Prunkl L, Vinella-Brusher E, Wang J, Oluyede L, Wolfe M (2022). Transportation barriers to care among frequent health care users during the COVID pandemic. BMC Public Health.

[22] Hircock C, Huan P, Pizzola C, McDonald M (2024). A scoping review of surgical care for people experiencing homelessness: prevalence, access, and disparities. Can J Surg.

[23] Decker H, Raguram M, Kanzaria HK, Duke M, Wick E (2024). Provider perceptions of challenges and facilitators to surgical care in unhoused patients: a qualitative analysis. Surgery.

[24] Clark NM, Hernandez AH, Bertalan MS (2025). Travel time as an indicator of poor access to care in surgical emergencies. JAMA Netw Open.

[25] Koster ES, Schmidt A, Philbert D, van de Garde EM, Bouvy ML (2017). Health literacy of patients admitted for elective surgery. Z Gesundh Wiss.

[26] De Oliveira GS Jr, McCarthy RJ, Wolf MS, Holl J (2015). The impact of health literacy in the care of surgical patients: a qualitative systematic review. BMC Surg.

[27] Garfinkel JH, Hummel A, Day J, Roney A, Jones M, Rosenbaum A, Ellis SJ (2020). Health literacy and recall of postoperative instructions in patients undergoing the Lapidus procedure. Foot Ankle Orthop.

[28] Seel M, Mihalic JA, Froschauer SM, Holzner B, Meier J, Gotterbarm T, Holzbauer M (2025). Changes in health education literacy after structured web-based education versus self-directed online information seeking in patients undergoing carpal tunnel release surgery: nonrandomized, controlled study. JMIR Form Res.

[29] Diallo MS, Hasnain-Wynia R, Vetter TR (2024). Social determinants of health and preoperative care. Anesthesiol Clin.

[30] Diallo MS (2024). Health equity and social determinants of health. Anesthesiol Clin.

[31] Luan-Erfe BM, DeCaria B, Tuncel C, Okocha O, Sweitzer BJ (2024). Survey of perioperative utilization of professional medical interpreters for limited-English proficient patients: towards a framework for systems-level improvement. Perioper Care Oper Room Manag.

[32] Wiles I, Tariq A, Karasneh G, Summerville D, Tovar DE, Woltenberg LN (2023). The effects of interpreter utilization on patient outcomes: a contemporary literature review. Educ Health Prof.

[33] Pires SM, Ramos A, Gomes I (2025). Exploring the emergent concept of patient advocacy in acute and perioperative settings: a scoping review. J Caring Sci.

[34] Patel J, Tolppa T, Biccard BM (2022). Perioperative care pathways in low- and lower-middle-income countries: systematic review and narrative synthesis. World J Surg.

[35] Choxi H, VanDerSchaaf H, Li Y, Morgan E (2022). Telehealth and the digital divide: identifying potential care gaps in video visit use. J Med Syst.

[36] Cheng J, Arora VM, Kappel N, Vollbrecht H, Meltzer DO, Press V (2023). Assessing disparities in video-telehealth use and eHealth literacy among hospitalized patients: cross-sectional observational study. JMIR Form Res.

[37] Martínez JL, Coca MÁM, Del Olmo Rodríguez M (2025). Effects of virtually led value-based preoperative assessment on safety, efficiency, and patient and professional satisfaction. J Clin Med.

[38] Malloy M, Tarima S, Canales B, Nelson D, Hanley J (2023). Identifying risk factors for appointment no-shows in a pediatric orthopaedic surgery clinic. J Pediatr Soc North Am.

[39] Theng B, Tran JT, Serag H, Raji M, Tzeng HM, Shih M, Lee WM (2023). Understanding caregiver challenges: a comprehensive exploration of available resources to alleviate caregiving burdens. Cureus.

[40] Jack K, Evans C, Bramley L, Cooper J, Keane T, Cope M, Hendron E (2022). Identifying and understanding the non-clinical impacts of delayed or cancelled surgery in order to inform prioritisation processes: a scoping review. Int J Environ Res Public Health.

[41] Thirunavukarasu GS, Partridge JS, Dhesi JK (2024). Addressing inequalities in the perioperative care for older adults. Br J Hosp Med (Lond).

[42] Tan H, Mendoza BA, Fortier MA, Kain Z (2022). Perioperative pain disparity in children: a call for action. Paediatr Anaesth.

[43] Martin SR, Kain ZN (2024). The intersection of pediatric anesthesiology and social determinants of health. Curr Opin Anaesthesiol.

[44] Wolfe K, Peyvandi S (2025). Neurodevelopmental outcomes in congenital heart disease: modifiable and nonmodifiable substrates. Curr Opin Cardiol.

[45] Mehta B, Goodman S, Ho K, Parks M, Ibrahim SA (2021). Community deprivation index and discharge destination after elective hip replacement. Arthritis Care Res (Hoboken).

[46] Elizabeth Baetzel A, Holman A, Dobija N, Reynolds PI, Nafiu O (2025). Racial disparities in pediatric anesthesia: an updated review. Anesthesiol Clin.

[47] Cyprich J, Pangal DJ, Rutkowski M (2021). Comparative preoperative characteristics and postoperative outcomes at a private versus a safety-net hospital following endoscopic endonasal transsphenoidal resection of pituitary adenomas. J Neurosurg.

[48] Wan YI, McGuckin D, Fowler AJ, Prowle JR, Pearse RM, Moonesinghe SR (2021). Socioeconomic deprivation and long-term outcomes after elective surgery: analysis of prospective data from two observational studies. Br J Anaesth.

[49] Gilman AT, Kim J, Jiang SY, Abramovitz SE, White RS (2025). Racial health disparities in severe maternal morbidity before and after implementation of an enhanced recovery after cesarean delivery protocol: a retrospective observational study at two New York City hospitals (2016-2020). Int J Obstet Anesth.

[50] Viveros-Carreño D, Agustí N, Rodríguez J, Mora-Soto N, Burbano J, Mayor A, Pareja R (2025). Inadvertent cervical cancer: a narrative review. Int J Gynecol Cancer.

[51] Thompson MP, Yost ML, Syrjamaki JD (2020). Sources of hospital variation in postacute care spending after cardiac surgery. Circ Cardiovasc Qual Outcomes.

[52] Fitzgerald DC, Simpson AN, Baker RA (2022). Determinants of hospital variability in perioperative red blood cell transfusions during coronary artery bypass graft surgery. J Thorac Cardiovasc Surg.

[53] Goldson KV, Brennan E, Burton BN (2025). Does management of postoperative nausea and vomiting differ by patient demographics? An evaluation of perioperative anesthetic management—an observational study. Anesthesiology.

[54] Ghasemi Shayan R, Fatollahzadeh Dizaji M, Sajjadian F (2025). Surgical and postoperative management of congenital heart disease: a systematic review of observational studies. Langenbecks Arch Surg.

[55] Chung IY, Benzy M, Kavitha S, Venkatesh R, Shekhawat N (2025). Travel and financial burdens of cataract surgical care in South India: comparison of postoperative follow-up at local vision centers versus an urban eye hospital. Indian J Ophthalmol.

[56] Stephens CQ, Butler MW, Samad L, Seyi-Olajide JO, Evans FM, Gathuya Z, McLeod E (2024). Children's surgery and the emergency, critical, and operative care resolution: immediate actions to eliminate disparities in surgery, anesthesia, and perioperative care for all children. Paediatr Anaesth.

[57] Bumin Aydın G, Sakızcı Uyar B (2021). Mothers level of education and preoperative informative story book reading helps reduce preoperative anxiety in children in Turkey. J Pediatr Nurs.

[58] Kim J, Sweitzer B (2025). Special considerations related to race, sex, gender, and socioeconomic status in the preoperative evaluation: part 1: race, history of incarceration, and health literacy. Anesthesiol Clin.

[59] Savage KT, Chen J, Schlenker K, Pugliano-Mauro M, Carroll BT (2025). Geriatric dermatologic surgery part II: peri- and intraoperative considerations in the geriatric dermatologic surgery patient. J Am Acad Dermatol.

[60] Soffin EM, Wilson LA, Liu J, Poeran J, Memtsoudis SG (2021). Association between sex and perioperative opioid prescribing for total joint arthroplasty: a retrospective population-based study. Br J Anaesth.

[61] Varley PR, Buchanan D, Bilderback A (2023). Association of routine preoperative frailty assessment with 1-year postoperative mortality. JAMA Surg.

[62] Dossabhoy SS, Manuel SR, Yawary F (2025). Implementation of a preoperative frailty screening and optimization pathway for vascular surgery patients is associated with decreased 30-day readmission. J Vasc Surg.

[63] Qin PP, Jin JY, Min S, Wang WJ, Shen YW (2022). Association between health literacy and enhanced recovery after surgery protocol adherence and postoperative outcomes among patients undergoing colorectal cancer surgery: a prospective cohort study. Anesth Analg.

[64] Gerlach RM, Sweitzer B (2025). Special considerations related to race, sex, gender, and socioeconomic status in the preoperative evaluation: part 2: sex considerations and homeless patients. Anesthesiol Clin.

[65] Robbins CB, Wisely CE, Rosdahl JA, Muir KW, Gupta D (2020). Impact of video education on patient knowledge, anxiety, and satisfaction in selective laser trabeculoplasty: a pilot study. J Glaucoma.

[66] Acker SN, Meraz AI, Wilson SN (2025). A randomized controlled trial of perioperative education via e-health technology to ensure high quality equitable care: One-4-All initiative. J Pediatr Surg.

[67] Kabbes N, Bugra A, Wissanji H, Osmanlliu E (2024). Telehealth for Indigenous children worldwide: a scoping review. J Pediatr Surg.

[68] Stewart H, Stanley S, Zhang X (2025). The inequalities and challenges of prehabilitation before cancer surgery: a narrative review. Anaesthesia.

[69] Le Sant G, Frouin A, Gachet L (2025). Effects of preoperative treatment on healthcare utilization and return to work for anterior cruciate ligament injuries: a real-world study using the French healthcare database. Phys Sportsmed.

[70] Barile F, Artioli E, Mazzotti A (2024). To cast or not to cast? Postoperative care of ankle fractures: a meta-analysis of randomized controlled trials. Musculoskelet Surg.

[71] Wu F, Liu P, Ou Z, Zhou S, Zhang C (2025). Early versus delayed weightbearing for postoperative ankle fractures: a systematic review and meta-analysis of randomized controlled trials. Arch Orthop Trauma Surg.

[72] Wilder FG, Bostock IC, Watkins A, Erfe JM, Erkmen C, Pereira S, Erhunmwunsee L (2025). Social determinants of health in thoracic surgery: critical points for understanding and implementing change. Ann Thorac Surg.

[73] Mergler BD, Toles AO, Alexander A, Mosquera DC, Lane-Fall MB, Ejiogu NI (2024). Racial and ethnic patient care disparities in anesthesiology: history, current state, and a way forward. Anesth Analg.

